# Brain MRI in Children with Mild Traumatic Brain Injury and Persistent Symptoms in Both Sports- and Non-sports-related Concussion

**DOI:** 10.7759/cureus.3937

**Published:** 2019-01-22

**Authors:** Alcy R Torres, Zakir I Shaikh, Wilson Chavez, John E Maldonado

**Affiliations:** 1 Pediatrics, Boston University School of Medicine, Boston, USA; 2 Pediatrics, Surat Municipal Institute of Medical Education and Research, Surat, IND; 3 Radiology, Boston Medical Center, Boston, USA; 4 Pediatrics, Pontifical Catholic University of Ecuador, Quito, ECU

**Keywords:** pediatric concussion, postconcussion syndrome, magnetic resonance imaging, mild traumatic brain injury, brain mri

## Abstract

Aim: To evaluate the utility of magnetic resonance imaging (MRI) in children with mild traumatic brain injury (mTBI), persistent post-concussive syndrome (PPCS), and normal neurologic examination.

Materials and methods: This was a retrospective review of pediatric patients, who were evaluated in a Pediatric Concussion Clinic between August 2013 and November 2018, with documented persistent post-concussive symptoms, normal neurological examination, and available brain MRI.

Results: In our analysis of 86 cases we found seven MRI studies with abnormal findings, but none were clinically significant.

Conclusion: We conclude that MRI has a low diagnostic yield in this population, and based on these results we recommend that clinicians should avoid ordering MRI studies in this group. Further research is necessary to validate these results in larger and prospective studies.

## Introduction

The aim of this study was to demonstrate the utility of brain magnetic resonance imaging (MRI) in children (aged 2–18 years) with mild traumatic brain injury (mTBI) and persistent post-concussive syndrome (>28 days) but with a normal neurological examination. We seek to identify abnormal brain imaging in these patients to assess the utility of neurological imaging in this group.

Traumatic brain injury (TBI) is one of the leading causes of hospital visits in the United States. In the last study cited by the Centers for Disease Control and Prevention (CDC) in 2013, there were approximately 640,000 TBI-related emergency department (ED) visits, 18,000 TBI-related hospitalizations, and 1,500 TBI-related deaths in children aged 0–14 years in the USA [1]. In 2012, approximately 325,000 ED visits resulted from sports and recreation-related mTBI [2]. Among the pediatric population with mTBI, 82% initially visited primary care, 5% searched for specialty care, and 12% were assisted at the ED [3,4].

TBI can be classified in a number of ways but traditionally the classification is based on clinical severity and duration of symptoms, and characteristics and location of the injury [5]. The severity of TBI is most commonly quantified by the Glasgow Coma Scale (GCS). A GCS score of 13–15 is considered mild injury, 9–12 is considered moderate injury, and eight or less is considered severe TBI [6].

There are different mechanisms of traumatic brain injury occurring in sports, war, and life. These mechanisms are closed head traumatic brain injury, blast-related brain injury, and penetrating brain injury. Those share many common features in the potentiation of injury through secondary injury mechanisms and physical and neuropsychological outcome. However, significant differences in pathophysiological pathways, gross neurologic damage, and the time scale of neurodegeneration exist, which have been studied using different methods [7].

Mild traumatic brain injury (mTBI) is defined as “an acute brain injury resulting from mechanical energy to the head from external physical forces”. The operational criteria for clinical identification include:

- One or more of the following: confusion or disorientation, loss of consciousness (LOC) for 30 minutes or less, post-traumatic amnesia for less than 24 hours, and/or other transient neurologic abnormalities such as focal signs, seizure, and intracranial lesion not requiring surgery.

- GCS score of 13-15 after 30 minutes post-injury or later upon presentation for health care.

- These manifestations of mTBI must not be due to drugs, alcohol, medications, caused by other injuries or treatment for other injuries (eg, systemic injuries, facial injuries, or intubation), caused by other problems (eg, psychological trauma, language barrier, or coexisting medical conditions), or caused by penetrating craniocerebral injury [8].

More than 75% of children with traumatic brain injuries attended to in the emergency department in the United States are defined as mild [9], but the numbers seeking care are rising dramatically [10] due to increased public awareness about concussion, improved training of clinicians in concussion diagnosis, and a legislation in all 50 states requiring healthcare provider clearance prior to returning to play [3,4]. Traumatic brain injury is the leading cause of death and disability in children older than one year [11]. The concerns about its effects on the developing brain have led to a large number of recent studies examining pediatric concussion [12]. Rivara et al. found that an approximate of 14% of children with mTBI experienced disability, defined as the use of specialized medical and educational services [13].

Fortunately, almost 90% of concussions are transient, with symptoms resolving within four weeks. However, a minority of patients remain symptomatic several months post injury [14]. The most common symptoms encountered at presentation are headaches, dizziness, and taking longer to think whereas sleep disturbance, frustration, forgetfulness, and fatigue are the symptoms most likely to develop during the follow-up period and that had not initially been present [12].

Symptoms persisting beyond 28 days are referred to as persistent post-concussion syndrome (PPCS) and can have serious adverse effects, resulting in school absenteeism, impaired academic performance, depressed mood, loss of social activities, and lower quality of life [15]. These symptomatic patients are usually the population seen in the clinic after the event, and the persistence of symptoms despite their normal neurological examination raises the concern of getting neuroimaging.

There have been some studies about brain imaging in patients with persistence of symptoms more than two weeks after a concussion, which showed low diagnostic yield and had poor cost-effectiveness. One such study, done in a smaller cohort and which included other different imaging modalities, determined that neuroimaging should not be repeated beyond the acute phase. Though head computed tomography (CT) had the relatively highest yield among the modalities, the potential effect of radiation exposure must be considered. MRI, a non-radiation method, can detect post-traumatic encephalomalacia, reactive gliosis, hemosiderin deposits, and microbleeds; however, these typically only accompany more severe forms of TBI [14].

Mild-to-moderate TBI-related services in children averaged $77.9 million per year in healthcare costs, with an average expenditure of $1,044 per capita in which $166 were TBI-specific services and $878 were for general healthcare [16].

## Materials and methods

We conducted a single-center retrospective review of electronic medical records and neuroimaging (MRI) studies for all patients with mild traumatic brain injury at Boston Medical Center from 2013 to 2018. The inclusion criteria were as follows: 1) age 2–18 years; 2) mTBI: GCS score of 13-15; and 3) normal neuro examination during ED evaluation after the episode and in a clinic a few days later. The exclusion criteria were as follows: 1) children with severe injury mechanisms defined by fall >0.9 m [3 feet]; head struck by high impact object; motor vehicle collision with patient ejection, death of another passenger, or rollover; pedestrian or bicyclist without helmet struck by a motorized vehicle; 2) penetrating trauma, known brain tumors, pre-existing neurological disorders complicating assessment; 3) pre-existing neuroimaging finding before event.

Data collection and analysis

Provider consultation notes, MRI findings, and scanned outside hospital documentation were collected and analyzed via our institution’s electronic medical record system. We collected data in three categories: 1) demographic variables, 2) past medical history, and 3) timing of MRI—only imaging obtained >four weeks after the event was included. A board-certified pediatric neurologist with the Symptom Evaluation of Children with the Sports Concussion Assessment Tool 5 (SCAT5©) determined clinically relevant findings [17]. A faculty neuroradiologist identified positive findings on MRI.

The analysis took into consideration any symptoms related to the trauma at the time of evaluation in the clinic after the event. Post-concussion symptoms were investigated using the symptom evaluation list of Children SCAT-3 & SCAT-5. Patients and their family responded to the questionnaire after the trauma.

All data was irreversibly made anonymous and this study was conducted according to the guidelines of the ethical committee of our institution. The institutional review board approved this study, with a waiver of informed consent. The study protocol complied with the Health Insurance Portability and Accountability Act.

## Results

Demographics

The mean age of all patients was 14.17 years with a range between six and 18 years (Table 1). The study included 46 females (53.49%) and 40 (46.51%) males. Most patients were of the 12–18 years age group.

**Table 1:** Demographic characteristics of participants

Table 1. Demographic characteristics of participants		
	Frequency	Percentage
Total (N)	86	100%
Age group		
2-5	0	0%
6-11	17	19.77%
12-18	69	80.23%
Age mean	14.17	
Number of patients	86	
Gender		
Male	40	46.51%
Female	46	53.49%

In all the patients with mild TBI, the most common symptom was headaches (88.37%), followed by memory, attention, and concentration disturbances (37.21%), and, lastly, visual disturbances (e.g. blurry vision, photosensitivity, photophobia) 36.05%. But, most of the children had more than one symptom (Table 2).

**Table 2:** Signs and symptoms in patients

Table 2. Signs and symptoms in patients		
Symptoms	Frequency	Percentage
Headaches	76	88.37%
Memory, attention, concentration disturbances	32	37.21%
Visual disturbances	31	36.05%
Dizziness	28	32.56%
Auditory disturbances	14	16.28%
Sleep disturbances	10	11.63%
Nausea	8	9.30%

Only 11 (12.79%) of the patients lost consciousness at the time of injury, five of them for less than one minute. Thirty-three (38.37%) of the patients had a sport-related concussion, where football was involved in most cases (seven) (8.14%), followed by martial arts (five cases) (5.81%). Fifty-three (61.63%) were not sport related, of which 17 (19.77%) were due to a motor vehicle accident, 17 (19.77%) were due to a fall, seven (8.14%) were due to a head strike, and seven (8.14) were due to an assault.

The most common mechanism of injury was direct impact (88.37%) as a result of a collision with other objects. Six (6.98%) of the patients had post-traumatic amnesia, of which one (1.16%) was anterograde, two (2.33%) were retrograde, and three (3.49%) had amnesia of the event.

The history of a prior concussion was recorded as: none (62.79%), one (24.42%), two (5.81%), three (2.33%), and more than four (4.65%). In our study, there was no relation between multiple concussions and abnormal MRI results. Most of the patients had an immediate onset (76.74%) of symptoms.

Some patients (75) (87.21%) responded to symptomatic treatment used for their most prominent symptoms, which include pharmacologic and non-pharmacologic therapies (Table 3).

**Table 3:** Clinical characteristics of patients

Table 3. Clinical characteristics of patients		
Characteristics	Frequency	Percentage
Type of injury		
Sport-related	33	38.37%
Football	7	8.14%
Martial arts	5	5.81%
Gymnastics	3	3.49%
Softball	3	3.49%
Cheerleading	2	2.33%
Non-sport related	53	61.63%
Motor vehicle accident	17	19.77%
Fall	17	19.77%
Head strike	7	8.14%
Assault	7	8.14%
Mechanism of injury		
Direct impact	76	88.37%
Acceleration/deceleration	6	6.98%
Not specified	4	4.65%
Loss of consciousness during event	11	12.79%
Average duration of loss of consciousness		
<1 minute	5	5.81%
>1 minute	6	6.98%
Post-traumatic amnesia	6	6.98%
About the event	3	3.49%
Retrograde	2	2.33%
Anterograde	1	1.16%
Prior concussion		
None	54	62.79%
1	21	24.42%
2	5	5.81%
3	2	2.33%
>4	4	4.65%
Prior neuro-psychologic disorder	39	45.35%
Attention deficit hyperactive disorder	12	13.95%
Depression	8	9.30%
Anxiety	6	6.98%
Onset of symptoms		
Immediate	66	76.74%
Gradual	20	23.26%
Response to symptomatic treatment		
Positive response	75	87.21%

Neuroimaging

Out of the 1275 patients who visited the clinic, 255 patients had PPCS, and MRI was obtained in 86 of these patients with PPCS (33.73%). Of these 86 patients, 14 (16.28%) had prior imaging in the acute phase that was negative and subsequently repeated during the chronic phase of their PPCS course. The indication to obtain the MRI was only prolonged persistent symptoms.

Among the 86 patients with MRI, seven patients had positive imaging findings, but none were clinically significant to alter the course or the medical management (Tables 4-5).

**Table 4:** Neuroimaging findings

Table 4. Neuroimaging findings		
Trauma-related	86	
Non-trauma-related	0	
MRI scan ordered post-concussion	86	
Timing of MRI after event	Frequency	Percentage
28 days - 3 months	32	37.21%
3 months - 6 months	29	33.72%
>6 months	25	29.07%

**Table 5:** Neuroimaging abnormalities

Table 5. Neuroimaging abnormalities	
Case	Findings
1	Irregularly shaped T2/FLAIR (T2-weighted and fluid-attenuated inversion recovery) hyperintensity areas in the right frontal subcortical white matter
2	Scattered tiny punctate regions of T2/FLAIR hyperintensity in the frontal lobes
3	Nonspecific foci of FLAIR hyperintensity in the left frontal lobe
4	Cluster of small cysts with mild surrounding gliosis in the right periatrial region
5	Nonspecific ovoid T2/FLAIR hyperintense focus within the left posterior temporal lobe
6	Atretic parietal cephalocele with a persistent falcine sinus and partial absence of the straight sinus
7	Numerous subcortical white matter FLAIR hyperintensities

## Discussion

In the span of five years, we evaluated 1275 concussion patients of whom 255 had PPCS. MRI was ordered in 86 (33.73%) patients but none had clinically significant findings. MRI studies require time, are expensive, use hospital resources, and might cause psychological stress for the patient and their family [16,18,19]. We conducted a retrospective study on this cohort to assess the utility of MRI and determine the need for its use.

In our study we found only seven (8.4%) abnormal MRI; however, none of the MRI had any clinical relevance. In those patients with positive findings, further diagnostic tests or interventional treatment were not required, and the management was not modified because of the imaging results. Requesting imaging in these patients did not add value and had a low diagnostic yield.

In 2015 Morgan et al. studied 19 patients who had MRI and only one had findings that were not considered clinically significant [14]. Although this series was very small, he concluded that brain imaging should only be ordered in very specific rare occasions. Results from our study are similar to the Morgan series and corroborate the low yield of MRI in children with persistent post-concussive symptoms. The abnormal MRI value that we found in our cohort is low as in the mentioned study [14]. In 2017 Rose et al. studied 134 patients who underwent MRI evaluations, and 16.4% were found to have incidental abnormalities, a rate slightly higher than the 10.6% cited among healthy youth [20]. Incidental findings can prompt additional imaging and another testing, further increasing healthcare costs. The imaging readings can also be a source of stress and anxiety for families. So, they conclude that a better understanding of the diagnostic yield and the therapeutic benefits of neuroimaging is needed to guide clinical decision-making when post-concussion symptoms persist. A similar study was done by Bonow et al., where a large database of sports concussion in the pediatric population with persistent symptoms was explored. They studied MRI reports of 427 pediatric patients and only 0.5% had findings compatible to the traumatic episode; 61 patients had abnormal MRI, but the findings were unrelated to trauma and without any clear clinical relevance. In comparison to the above-mentioned studies, our research was different as it focused on both sports- and non-sports-related concussions and included only those patients with a normal neurological examination [21].

The estimated average list price for a single brain MRI nationwide was $2625 per study. Among the 86 patients with PPCS who received MRIs, we estimated a total billed cost of $225,750. The cost of one positive MRI finding was $32,250. We used New Choice Health for the estimated cost of brain MRI [22], a resource that has been cited in previous literature.

MRI has equal sensitivity in detecting acute changes and better sensitivity than head CT in detecting some anomalies, such as cerebral edema, hemosiderin deposition, and minor cerebral contusion, and avoids the substantial radiation exposure that accompanies head CT [23]. Brain MRI can detect subtle signs of traumatic injury that may influence concussion management decisions when symptoms persist (e.g. return to play and retirement from a high-risk sport), yet a correlation between MRI findings and clinical outcomes has not been well-established. Scanty data exists about the clinical utility of MRI in patients with persistent post-concussion symptoms and the purpose of our study is to contribute more information about this controversial topic in children with mild traumatic brain injury and persistent symptoms in both sports- and non-sports-related concussion.

When present on clinic day MRI appears to have been the preferred imaging modality for late-acute and sub-acute cephalalgic symptoms. In our study, MRI was the method of choice to further evaluate patients with persistent post-concussive syndrome. CT is generally performed during the acute post-concussion period, while the mean time to MRI is nearly 40 days following injury in our study and others.

There may also be the possibility to predict the development of persistent post-concussion symptoms in children presenting to the emergency department, although a clinical score developed had modest discrimination to estimate persistent post-concussive syndrome risk in those patients. However, features such as female sex and older age are associated with prolonged recovery in children and adults [15].

Following the Pediatric Emergency Care Applied Research Network (PECARN) rules that guide the decisions in the ED in children with mTBI, a study found that doctor visits following mild TBI were significantly more frequent within patients from the hospitalized group than the ambulatory group [24].

The positive findings on MRI in our population do not explain the persistence of symptoms. However, a study shows that parenchymal lesion on MRI can reflect greater risk for brain insult and predict higher levels of PPCS within the mTBI [25]. Our results are probably not related because we did not find major neurological imaging changes that can alter the course of the recovery from a mild traumatic brain injury but more importantly because our subjects had normal neurological examinations. In addition to clinic day symptoms, prior concussions and continued active participation at the time of injury predicted MRI utilization.

It is also important to consider the psychological factors when deciding to order further studies in a patient with mTBI and post-concussive symptoms. Studies have suggested that psychological factors may play some role, whereby obtaining CT or MRI scans changed the patients' perceptions about their injuries and led to longer symptom recoveries [20].

The limitation of the study, in general, is its retrospective design. There is also the fact that we recorded the post-concussion symptoms using a questionnaire, leading to subjective information.

## Conclusions

In our population of pediatric patients with a history of sports- and non-sports-related concussion and post-concussive syndrome, a brain MRI was of low yield diagnosis if they had a normal neurologic examination. Further research is necessary to validate our results in larger and prospective studies.
